# Evaluation of clustering algorithms for gene expression data

**DOI:** 10.1186/1471-2105-7-S4-S17

**Published:** 2006-12-12

**Authors:** Susmita Datta, Somnath Datta

**Affiliations:** 1 Department of Bioinformatics and Biostatistics, University of Louisville, Louisville, KY 40202, USA

## Abstract

**Background:**

Cluster analysis is an integral part of high dimensional data analysis. In the context of large scale gene expression data, a filtered set of genes are grouped together according to their expression profiles using one of numerous clustering algorithms that exist in the statistics and machine learning literature. A closely related problem is that of selecting a clustering algorithm that is "optimal" in some sense from a rather impressive list of clustering algorithms that currently exist.

**Results:**

In this paper, we propose two validation measures each with two parts: one measuring the statistical consistency (stability) of the clusters produced and the other representing their biological functional congruence. Smaller values of these indices indicate better performance for a clustering algorithm. We illustrate this approach using two case studies with publicly available gene expression data sets: one involving a SAGE data of breast cancer patients and the other involving a time course cDNA microarray data on yeast. Six well known clustering algorithms UPGMA, K-Means, Diana, Fanny, Model-Based and SOM were evaluated.

**Conclusion:**

No single clustering algorithm may be best suited for clustering genes into functional groups via expression profiles for all data sets. The validation measures introduced in this paper can aid in the selection of an optimal algorithm, for a given data set, from a collection of available clustering algorithms.

## Background

Cluster analysis is an exploratory technique that might reveal classes or groups of genes that act in consort during a biological process. A distance or dissimilarity is calculated between the expression vectors of each pair of genes. A statistical clustering algorithm is then employed which places a pair of genes in the same cluster if their expression profiles are similar as judged by the distance measure employed. The exact details of achieving this goal varies from one algorithm to the next. In addition, more complex and relatively modern algorithms offer the users with several choices of tuning parameters. The resulting grouping may be quite varied (see, e.g., [1]; [2,3]).

The problem of selecting the "best" algorithm/parameter setting is a difficult one. A good clustering algorithm ideally should produce groups with distinct non-overlapping boundaries, although a perfect separation can not typically be achieved in practice. Figure of merit measures (indices) such as the silhouette width [4] or the homogeneity index [5] can be used to evaluate the quality of separation obtained using a clustering algorithm. The concept of stability of a clustering algorithm was considered in [3] (also see [6]). The idea behind this validation approach is that an algorithm should be rewarded for consistency. They compared the results of clustering with the full data and the reduced data after reducing the expression profiles by one unit. In this paper we provide two case study examples where we evaluate the relative performances of six well known algorithms. In doing so, we introduce two new measures to judge the quality of the clusters using the existing biological knowledge about the genes from ontology databases. We also look at their overall performance by combining these measures with their statistical consistency or stability. A detailed study of ten clustering algorithms using two other biological performance measures was recently published by us [7].

From a rather extensive range of existing clustering algorithms we select six representative algorithms from various groups each representing a different underlying principle. This list includes the popular hierarchical clustering where two smaller groups are joined to form a bigger cluster based on their average pairwise correlation. This is also known as UPGMA (Unweighted Pair Group Method with Arithmetic mean) and is perhaps the most commonly used clustering in the microarray context. We also include the most common partition method called the K-means algorithm [8], a divisive clustering method Diana, a fuzzy logic based method Fanny, a very popular neural network based method SOM (self-organizing maps, [9]) and a statistical method known as Model Based clustering. Most of these methods are described in [10]. See [11] for S+ or R implementations.

## Results

First we consider the expression profiles of 258 significant genes based on their 11 dimensional expression profiles over four normal and seven DCIS samples [12]. See the Methods section for further description of this data set. Based on the size of the data set and given that there are at least three functional classes we judge that a cluster size between four and eight might be appropriate.

**Figure 1:**
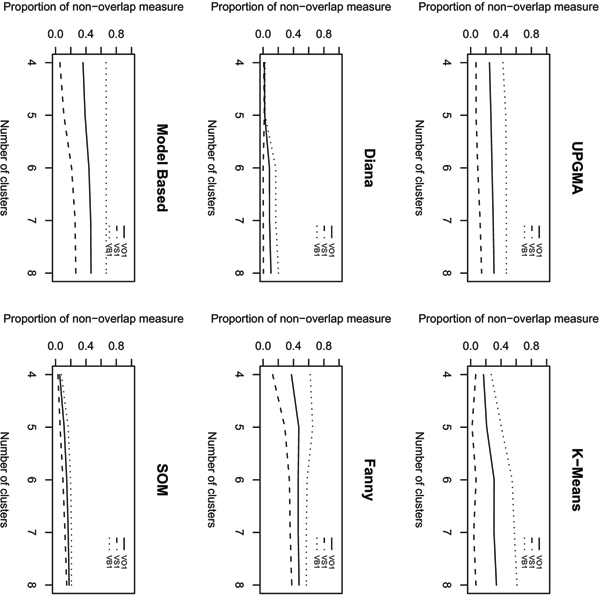
Proportion of non-overlap measures for various clustering algorithms applied to the normal and DCIS samples in breast cancer data. The dashed lines denote the statistical component, the dotted lines denote the biological component and the solid lines denote the overall (average) measure.

**Figure 2:**
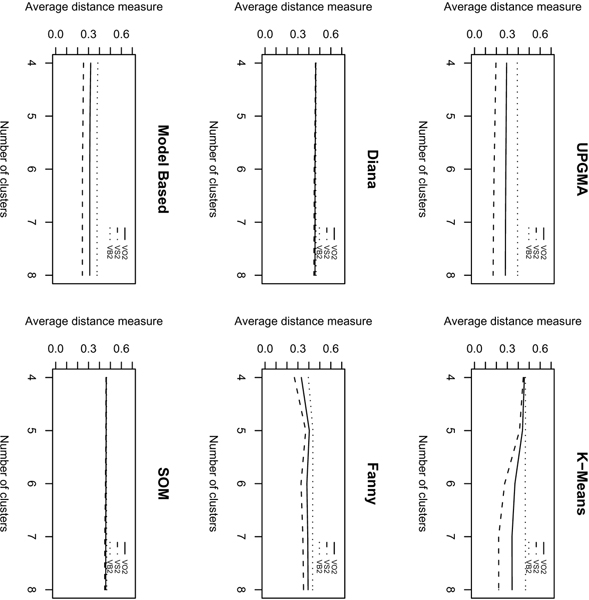
Average distance measures for various clustering algorithms applied to the normal and DCIS samples in breast cancer data. The dashed lines denote the statistical component, the dotted lines denote the biological component and the solid lines denote the overall (average) measure.

Figure 1 displays a panel of plots for the overall "proportion of non-overlap" validation measure *V*_*O*,1 _(5), with equal weights for the statistical and biological components, for the above clustering algorithms. Each plot also shows the two components separately, with dashed lines displaying the statistical component and the dotted lines plotting the biological component respectively. Clearly, based on these plots, we can say that Diana appears to be the best performer for this data set and SOM is a close runner-up. In addition, UPGMA is also performing reasonably well. Next we focus to the second performance measure *V*_*O*,2_. We have used 0.5(1 - *corr*) as the "distance" for computing this measure, where *corr *is the Pearson's correlation coefficient between the expression vectors. The corresponding panel plots are shown in Figure 2. The performance of both Diana and SOM are similar. However, they are no longer the best as judged by the average distance measure. With respect to this measure, the best performers are UPGMA and Model Based. Thus, overall, UPGMA appears to be a good performer for this data set as judged by two very different validation measures.

**Figure 3:**
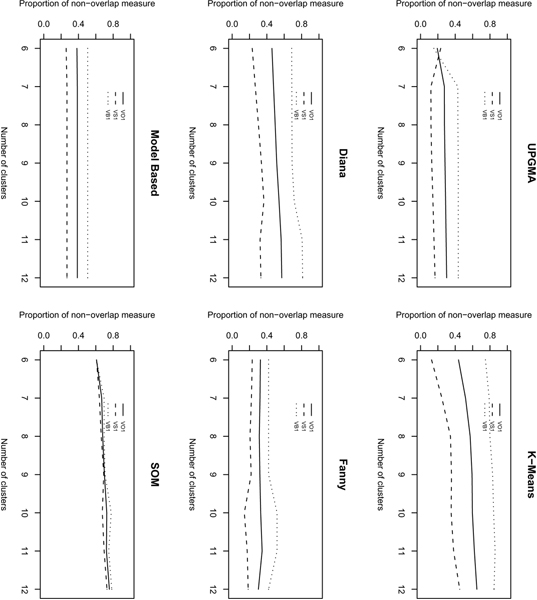
Proportion of non-overlap measures for various clustering algorithms applied to the yeast sporulation data. The dashed lines denote the statistical component, the dotted lines denote the biological component and the solid lines denote the overall (average) measure.

**Figure 4:**
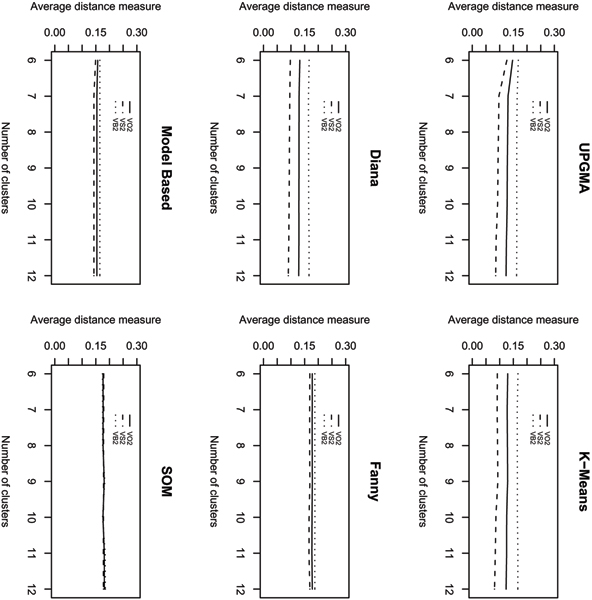
Average distance measures for various clustering algorithms applied to the yeast sporulation data. The dashed lines denote the statistical component, the dotted lines denote the biological component and the solid lines denote the overall (average) measure.

Next, we report the results for the yeast data [13]. Figures 3 and 4 provide the panel plots for the six clustering algorithms under consideration with respect to the two validation measures. As seen from Figure 3, both UPGMA and Fanny appear to be the best performers here as judged by each of statistical and biological components as well as the overall measure – a finding consistent with our earlier results for this data set [3]. Model based clustering also performs fairly well – which appears to be contradictory to our earlier reports [3]. This is presumably due to the fact that the current version of mclust in R is different from the earlier S-Plus version employed in that paper. UPGMA continues to be a solid performer even with respect to the second validation measure (Figure 4). The performances of Diana and K-Means also appear to be amongst the best with respect to this measure. Note that the "distance" *d *considered here for computing this validation measure is different from our earlier study [3].

## Discussion

We introduce a novel approach of combing both statistical consistency and biological congruence of the clusters produced by a clustering method. Two validation measures are proposed that are averages of two parts measuring statistical stability and biological congruence, respectively. A training (annotated) set of genes with known biological functions are used to judge biological congruence.

Our validation measures are easy to interpret and straightforward to compute. Graphs of these measures over a range of *k *(number of clusters) show the relative performance of a clustering algorithm. While there may not be a clear winner in all cases, this certainly represents a systematic approach in searching for the right algorithm for a data set amongst a collection of well known clustering algorithms, all of which are generally regarded as good algorithms.

The data examples used in this paper show that a clustering algorithm should be scrutinized from various angles. Certainly, the cross examinations using the two validation measures often showed different strengths and weaknesses of a clustering algorithm.

## Conclusion

No single clustering algorithm may be best suited for clustering genes into functional groups via expression profiles for all data sets. The validation measures introduced in this paper can aid in the selection of an optimal algorithm, for a given data set, from a collection of clustering algorithms. Whereas, the best algorithm in each case depends on which validation measure we employ, the performance of UPGMA appeared to be robust in both case studies undertaken in this paper.

## Methods

We denote by  the set of all genes for a given microarray experiment. Suppose the functional roles (e.g., biological functions) of a subset  of genes are known using an existing ontology database (e.g., Gene Ontology, Locus Link, Unigene cluster). Let's assume each gene in  belongs to one or more of the *F *functional classes _1_, ...., _*F*_. We provide two such examples later in the Methods section.

Consider two genes *x, y *that belong to the same functional class. Let us say that _*x *_is the statistical cluster containing gene *x*. Similarly _*y *_contains gene *y*. As genes *x *and *y *are in the same functional group we expect the two statistical clusters to be the same. We provide the following mathematical measure to evaluate the biological congruence of the statistical clusters:



where for a set *A, n*(*A*) denotes its size or cardinality. This measure is different from that proposed in [7] or [14]. This measure can be regarded as an average proportion of unequal statistical clusters containing gene pairs with similar biological functions. Simple measures similar to this have been used in the context of measuring accuracy of gene trees (see, e.g., [15]).

We also consider a second measure representing average distance between statistical clusters containing gene pairs with similar biological functions defined as



where *d*(*g*, *g*') is a distance or dissimilarity (e.g., Euclidean, Manhattan, 1-correlation, etc.) between the expression profiles of genes *g *and *g*'.

Next we capture the statistical validation of a clustering algorithm by inspecting the stability of the clusters produced when the expression profile is reduced by one observational unit. Using this idea the following two validation measures *V*_*S*,1 _and *V*_*S*,2_were proposed in [3] to measure statistical consistency.

In a microarray study, each gene has an expression profile that can be thought of as a multivariate data value in ℜ^*p*^, for some *p *> 1. For example, in a time course microarray study, *p *could be the number of time points at which expression readouts were taken. In a two sample comparison, *p *could be the total (pooled) sample size, and so on. For each *i *= 1, 2, ... , *p*, repeat the clustering algorithm for each of the *p *data sets in ℜ^*p*-1 ^obtained by deleting the observations at the *i*th position of the expression profile vectors. For each gene *g*, let ^*g, i *^denote the cluster containing gene *g *in the clustering based on the reduced expression profile. Let ^*g*,0 ^be the cluster containing gene *g *using the full expression profile. The following stability measures were introduced in [3]. The first measure is given by



This measure computes the (average) proportion of genes that are common to matched clusters on the basis of the full profile and the reduced profile obtained by deleting a single expression level. The second statistical validation measure we consider is



where *d*(*g*, *g*') is as before. This measure computes the average distance between the expression levels of all genes in matched clusters obtained on the basis of the full profile and the reduced profile, respectively.

Our final validation measure of a clustering algorithm is an average of the two parts representing biological congruence and statistical stability:

*V*_*O*,*l *_= (*V*_*B*,*l *_+ *V*_*S*,*l*_)/2, *l *= 1,2;     (5)

or



Note that (6) is equivalent to averaging in the log-scale. As before, a good clustering algorithm would yield a relatively small value of *V*_*O*,*l*_.

### Human breast cancer progression data

We illustrate our methods using the expression profiles of 258 genes (SAGE tags) that were judged to be significantly differentially expressed at 5% significance level between four normal and seven ductal carcinoma in situ (DCIS) samples [12]. [12] combined various normal and tumor SAGE libraries in the public domain with their own SAGE libraries and used a modified form of *t*-statistics to compute *p*-values. Further details can be obtained from their paper and its supplementary web-site.

Functional classes were constructed using a publicly available web-tool called Amigo [16]. A total of 113 SAGE tags were annotated into the following eleven functional classes based on their primary biological functions: cell organization and biogenesis (24), transport (7), cell communication (15), cellular metabolism (48), cell cycle (6), cell motility (7), immune response (7), cell death(7), development (5), cell differentiation (5), cell proliferation (5), where the numbers in parentheses were the numbers of SAGE tags in a class. As indicated earlier, some of the genes fell under multiple categories.

### Yeast sporulation data

We consider the yeast sporulation data set collected by [13]. This data set has expression levels of yeast genes during a time course sporulation experiment recorded at seven time points. The data set was filtered using the same criterion as in the original paper [13] to restrict to the genes whose expression levels showed significant changes during the course of the experiment. For our illustration, we look at a further subset of 513 genes (ORF's to be correct) that were overall positively expressed (for which, ∑_*time *_log expression ratio > 0). We annotated 503 of the 513 genes using the web-based GO mining tool FunCat [17] at [18]. They were placed into seventeen overlapping functional classes: metabolism (138), energy (27), cell cycle and DNA processing (152), transcription (50), protein synthesis (10), protein fate (72), protein with binding function or cofactor requirement (81), protein activity regulation (16), transport (63), cell communication (12), defense (36), interaction with environment (33), cell fate (17), development (41), biogenesis (77), cell differentiation (82).

### The clustering algorithms

For the illustrations and case studies, we have selected six well known clustering algorithms representing the vast spectrum of clustering techniques that are available in statistical pattern recognition and machine learning literature. All of them are validated using each of the two overall validation measures (5) with equal weights between statistical and functional validation.

#### UPGMA

This is perhaps the most commonly used clustering method with microarray data sets. This algorithm produces a tree (dendrogram) representing a hierarchy of clusters in an agglomerative manner. At each stage (level), two smaller clusters that are judged to be the closest based on their average pairwise correlation measure are joined together to form a bigger cluster. The tree can be cut at a chosen height to produce the desired number of clusters.

#### K-Means

This is a representative of the partition based algorithms where the number of clusters needs to be fixed in advance. It uses a minimum "within-class sum of squares from the centers" criterion to select the clusters. See [8] for further details.

#### Diana

This is a representative of a divisive clustering algorithm which produces a tree of clusters at the end. As the name suggests, at each stage a bigger cluster is divided into two smaller clusters following an optimization criterion.

#### Fanny

This algorithm produces a fuzzy cluster which is represented by a probability vector for each observation. The probabilities estimate its chances of belonging to the various clusters. A hard cluster assignment can be made by placing an observation to a cluster for which this estimated probability is the highest. A possible downside is that this may produce fewer hard clusters than desired.

#### Model Based

This is based on fitting a statistical model (mixtures of Gaussian distributions) to the data. Generally, a cluster membership is regarded as an unknown parameter which is estimated along with other distributional parameters via the method of maximum likelihood. See [19] for further details. Once again, this algorithm may produce less than the desired number of clusters which represent the number of mixture components in the data.

#### SOM

This is a member of a neural network based clustering. SOM stands for self-organizing maps [9]. It is a very popular method amongst the computational biologists and machine learning researchers.

## Authors' contributions

Susmita Datta: Development of statistical methods, identification of data sets, biological commentary and manuscript preparation; Somnath Datta: Development of statistical methods and computing and manuscript preparation. Both authors approved the final manuscript.

## References

[B1] Quackenbush J (2001). Computational analysis of microarray data. Nat Rev Genet.

[B2] Datta S, Arnold J, Gulati C, Lin YX, Mishra S, Rayner J (2002). Some comparisons of clustering and classification techniques applied to transcriptional profiling data. Advances in Statistics, Combinatorics and Related Areas.

[B3] Datta S, Datta S (2003). Comparisons and validation of statistical clustering techniques for microarray gene expression data. Bioinformatics.

[B4] Rousseeuw PJ (1987). Silhouettes: a graphical aid to the interpretation and validation of cluster analysis. J Computat Appl Math.

[B5] Shamir R, Sharan R (2002). Algorithmic approaches to clustering gene expression data. Current Topics in Computational Molecular Biology.

[B6] Dudoit S, Fridlyand J (2002). A prediction-based resampling method to estimate the number of clusters in a dataset. Genome Biol.

[B7] Datta S, Datta S (2006). Methods for evaluating clustering algorithms for gene expression data using a reference set of functional classes. BMC Bioinformatics.

[B8] Hartigan JA, Wong MA (1979). A k-means clustering algorithm. Applied Statistics.

[B9] Kohonen T (1997). Self-Organizing Maps.

[B10] Kaufman L, Rousseeuw PJ (1990). Finding Groups in Data. An Introduction to Cluster Analysis.

[B11] Venables WN, Ripley BD (1998). Modern Applied Statistics with S-Plus.

[B12] Abba MC, Drake JA, Hawkins KA, Hu Y, Sun H, Notcovich C, Gaddis S, Sahin A, Baggerly K, Aldaz CM (2004). Transcriptomic changes in human breast cancer progression as determined by serial analysis of gene expression. BMC Bioinformatics.

[B13] Chu S, DeRisi J, Eisen M, Mulholland J, Botstein D, Brown PO, Herskowitz I (1998). The Transcriptional Program of Sporulation in Budding Yeast. Science.

[B14] Gat-Viks I, Sharan R, Shamir R (2003). Scoring clustering solutions by their biological relevance. Bioinformatics.

[B15] Taylor JT, Piel WH (2004). An assessment of accuracy, error and conflict with support values from genome-scale phylogenetic data. Mol Biol Evol.

[B16] AmiGO. http://www.godatabase.org/cgi-bin/amigo/go.cgi.

[B17] Ruepp A, Zollner A, Maier D, Albermann K, Hani J, Mokrejs M, Tetko I, Güldener U, Mannhaupt G, Münsterkötter M, Mewes HW (2004). The FunCat, a functional annotation scheme for systematic classification of proteins from whole genomes. Nucleic Acids Research.

[B18] MIPS Functional Catalogue. http://mips.gsf.de/proj/funcatDB/search_main_frame.html.

[B19] Banfield JD, Raftery AE (1993). Model-based Gaussian and non-Gaussian clustering. Biometrics.

